# VoICE: A semi-automated pipeline for standardizing vocal analysis across models

**DOI:** 10.1038/srep10237

**Published:** 2015-05-28

**Authors:** Zachary D. Burkett, Nancy F. Day, Olga Peñagarikano, Daniel H. Geschwind, Stephanie A. White

**Affiliations:** 1 Department of Integrative Biology & Physiology, University of California, Los Angeles, California 90095; 2 Interdepartmental Program in Molecular, Cellular, & Integrative Physiology, University of California, Los Angeles, California 90095; 3 Program in Neurogenetics, Department of Neurology, David Geffen School of Medicine, University of California, Los Angeles, California 90095; 4 Center for Autism Research & Treatment, Semel Institute for Neuroscience & Human Behavior, University of California, Los Angeles, California 90095; 5 Center for Neurobehavioral Genetics, Semel Institute for Neuroscience & Human Behavior, University of California, Los Angeles, California 90095.

## Abstract

The study of vocal communication in animal models provides key insight to the neurogenetic basis for speech and communication disorders. Current methods for vocal analysis suffer from a lack of standardization, creating ambiguity in cross-laboratory and cross-species comparisons. Here, we present VoICE (Vocal Inventory Clustering Engine), an approach to grouping vocal elements by creating a high dimensionality dataset through scoring spectral similarity between all vocalizations within a recording session. This dataset is then subjected to hierarchical clustering, generating a dendrogram that is pruned into meaningful vocalization “types” by an automated algorithm. When applied to birdsong, a key model for vocal learning, VoICE captures the known deterioration in acoustic properties that follows deafening, including altered sequencing. In a mammalian neurodevelopmental model, we uncover a reduced vocal repertoire of mice lacking the autism susceptibility gene, *Cntnap2.* VoICE will be useful to the scientific community as it can standardize vocalization analyses across species and laboratories.

Though no animal model adequately captures the sophistication of language, ethological study of vocal communication has yielded valuable insight to its evolution and physiological basis. The learned songs of oscine songbirds are well-studied in the laboratory environment. The discrete brain circuitry, shared molecular reliance with humans, requirement of auditory feedback for maintenance, and parallel anatomical loops for producing learned vocalizations have made songbirds a powerful model for speech and language^1^,^2^. A key strength of rodent model systems is their genetic tractability, allowing researchers to precisely manipulate potential disease genes or neural circuits. In contrast to birdsongs, the ultrasonic vocalizations (USVs) generated by rodents are largely innate yet none-the-less provide an important phenotypic dimension^3^,^4^. As interest in a comprehensive analysis of social communication signals increases, the need for standardization across models becomes apparent. To meet this challenge, we designed an analysis pipeline into which any type of discrete vocal element (VE) can be input, and the output of which provides valid results in both acoustic and syntactical (defined here as the sequence in which vocal elements occur) domains.

The learned courtship song of male zebra finches (*Taeniopygia guttata*) exists in the audible range (20 Hz to 20 kHz), and is hierarchically composed of notes, syllables, motifs, and bouts^5^. Notes, the smallest unit, are defined as a region of a syllable that maintains a temporally continuous frequency pattern. Syllables are discrete vocal units that contain one or more notes. Motifs are repeated units of 3 or more syllables lasting ~1 second. A bout of song is composed of multiple motifs, frequently with repeated introductory syllables at the beginning of each bout. Analytical methods for quantifying the acoustic and syntactical components of song^6^,^7^,^8^,^9^,^10^ have proven efficacious, but their cross-species application is limited. Moreover, they exhibit a combination of drawbacks, including low resolution in quantifying acoustic differences between syllables due to calculating distance in a limited acoustic feature space, and/or a low throughput due to extensive user parsing of the data. Lastly, methods for quantifying syntax require the user to explicitly dictate the number of syllable types that emerge from the analysis or to incorporate the acoustic domain into the calculation of sequential similarity.

Mouse (*Mus musculus*) USVs occupy a frequency range that extends above 20 kHz and are generated in a variety of contexts. For example, when neonates become separated from the dam, they emit retrieval calls. Adult male mice make courtship calls when presented with females or their pheromones, whereas females produce calls when searching for their pups or in the presence of another female^11^. USVs are mostly narrowband, with spectral power distributed across a limited frequency range. In contrast to the stereotyped adult songs of zebra finches, a high degree of call-to-call variability exists in the mouse ultrasonic repertoire. Despite this variability, 10 distinct retrieval call categories have been defined and adopted (or modified), allowing for quantitative analyses of vocal signals generated by neonatal mice^12^. The process of individually parsing these calls into one of the categories, however, is labor-intensive, requiring manual inspection of each call and a subjective assignment into a category. This strategy leads to poor reproducibility of classifications, reduces the throughput of the analysis, and provides numerous opportunities for error. Efforts to automate this procedure resulted in the empirical derivation of a considerably reduced number of distinct call types, whose broad adoption would require a complete shift in how researchers consider the extent of the mouse vocal repertoire in order to maintain comparable and reproducible results across laboratories^13^.

To circumvent the limitations of existing vocal analysis techniques in both songbirds and rodents, we developed VoICE, software that utilizes acoustic similarity relationships between VEs to generate high dimensional similarity matrices, which are then input to a hierarchical clustering algorithm. We glean distinct syllable or call “types” by using an algorithm that automatically prunes clusters from hierarchical trees^14^. MATLAB is utilized for the user interface and similarity scoring, while scripts written in the R statistical programming language perform clustering and dendrogram trimming. We validate VoICE by applying it to vocalizations from adult male zebra finches and to mouse pups, and then compare the results to those obtained through manual parsing of finch syllables or mouse calls. We then illustrate the utility of VoICE by quantifying the phonological (i.e. acoustic) and syntactical consequences of deafening in the zebra finch. Further, use of VoICE replicates the finding of reduced numbers of retrieval calls in pups lacking the *Cntnap2* gene, an established model of autism^15^, and also uncovers changes in the repertoire of these animals. These findings establish this approach as a reliable, high-throughput method that faithfully captures known features of avian and rodent vocalizations and is capable of uncovering novel changes in this critical phenotypic trait.

## Results

### Overview: Semi-automated clustering of vocalizations

We present a method for the semi-automatic clustering of finch song syllables and mouse USVs through hierarchical clustering and automated dendrogram trimming. VEs in the form of zebra finch song syllables or mouse pup ultrasonic calls, were scored against themselves in a pairwise fashion to determine their acoustic similarity (Methods). The dimensionality of the resulting similarity matrix is limited only by the number of VEs that were recorded and used for input. This high degree of dimensionality provides greater specificity in grouping similar vocalizations, as compared to when clusters are based only on a finite number of acoustic features. The spectral co-similarity relationships between syllables are next subjected to hierarchical clustering, to generate a dendrogram, which is then trimmed into clusters using an automated tree-pruning algorithm. Originally developed for gene coexpression analyses, this tree-trimming algorithm has repeatedly yielded biologically meaningful clusters of genes from hierarchical trees^14^. Key advantages over other clustering methods include that the number of clusters (in this case, syllable or call types) is not dictated by the experimenter, providing for unbiased calculation of vocal repertoire. Following pruning of the dendrogram and determination of the number of syllable or call types, acoustic data for vocalizations of the same type is compiled and a syntax is generated. Vocalizations from subsequent recording sessions can then be compared to existing clusters, enabling both phonological and syntactical assessments across time, experimenters, laboratories, strains, genotypes or any other condition.

### Validation of VoICE in birds

**Figure 1:**
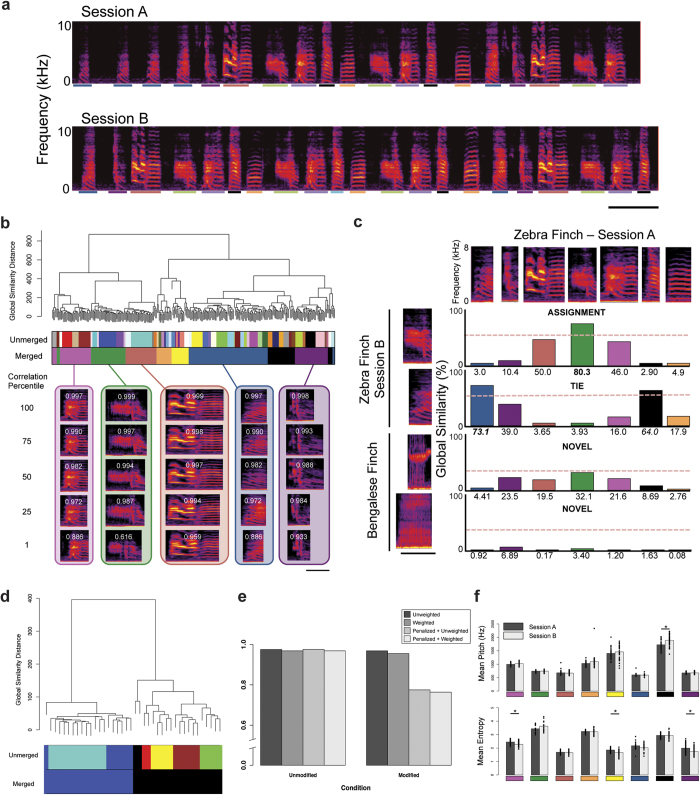
Assignment and quantification of clustered birdsong syllables. (**a**) Mature zebra finches (>120d) sing stereotyped song composed of repeated syllables that form motifs that form bouts. Shown are two song bouts sung by the same adult bird during two recording epochs (‘Session A’ and ‘Session B’). (Scale bar = 250 msec.) (**b**) Dendrogram plots global similarity distance between leaves (syllables) and was generated following spectral similarity scoring. Beneath the branches, clusters before (Unmerged) and after merging (Merged) are denoted by color bands. Representative syllables from merged clusters are illustrated at descending percentiles following correlation of each cluster member to the cluster eigensyllable. The Pearson’s rho for the correlation between each syllable and its eigensyllable are displayed in white. (**c**) During assignment, one of three possible outcomes for each syllable occurs: automatic assignment to a cluster (ASSIGNMENT), manual assignment in a tiebreaking procedure when statistically similar to two clusters (TIE), or categorization as novel (NOVEL). Artificially introduced syllables from a Bengalese finch did not pass a global similarity floor and are accurately deemed ‘novel’. Bars indicate the mean percentage global similarity between the syllable and each cluster. (**d**) The two artificially introduced syllables from a Bengalese finch, are, upon merging (Merged), appropriately assigned to two novel clusters. (**e**) Syntaxes are highly similar between recording sessions, regardless of metric used for scoring (left, ‘unmodified’) but the artificial introduction of novel syllables to the second recording session reduces similarity when using a metric that penalizes for novel syllables (right, ‘modified’). (**f**) Pitch (top) and entropy (bottom) are largely unchanged between recording sessions. (* = p < 0.05, resampling independent mean differences. Cluster colors are consistent throughout. Scale bars = 50 msec.)

Zebra finch songs consist of multiple syllables that are repeated in a specific pattern to form motifs, the neuroethologically relevant unit of a song^16^ (Fig. 1a). To validate VoICE in birdsong analysis, we examined the first ~300 syllables sung on two separate days, seven days apart. ‘Session A’ comprised 308 syllables and ‘Session B’ comprised 310. Due to the stereotyped nature of adult song, we predicted that songs would retain their phonology and syntax over time; an outcome that would support the utility of VoICE. Syllables from the Session A were extracted using the “Explore and Score” module of Sound Analysis Pro^8^ (SAP). Similarity scores between all syllables were calculated (Fig. S1) and the resultant similarity matrix was imported and hierarchically clustered in R, resulting in the production of a dendrogram. The algorithm produced 54 unique clusters, which were merged to 8 final clusters by a guided procedure (Methods, Supplementary Note 1), each representing a syllable in the motif (Fig. 1b). For each cluster, an ‘eigensyllable’ was calculated to represent the syllable that best describes the variance within the cluster (Methods). The syllables in each cluster were correlated to the eigensyllable and ranked to determine overall homogeneity in the cluster. The syllable with the lowest correlation to the eigensyllable was visually inspected to ensure that all syllables were properly assigned to each cluster. The average correlation of the lowest ranked syllable to the eigensyllable across all clusters was 0.788, which captures the stereotypy of adult birdsong.

To test the expectation that phonology and syntax would be equivalent between the two sessions, syllables from Session B were assigned to clusters representing the Session A syllable types using a global similarity floor of 60 (Fig. 1c). Syllables from Session B were assigned to Session A clusters using one of three possible outcomes: 1) 291 syllables were algorithmically determined to belong to a specific cluster (‘assignment’) resulting from a pairwise-comparisons post-hoc test following a statistically significant ANOVA result (p < 0.05) for all clusters exceeding the global similarity floor; 2) 19 syllables were manually assigned to a cluster in the case of a non-significant ANOVA result; and 3) 0 syllables were deemed ‘novel’. To test the validity of the novel syllable classification, 20 renditions of two syllables from a different songbird species, the Bengalese finch (*Lonchura striata domestica*), were subjected to the assignment procedure. As predicted, the 40 Bengalese finch syllables were deemed ‘novel’ using a GS floor of 40 and appropriately assigned to two new clusters (Fig. 1c–d).

To compare sequential similarity between Sessions A and B, four syntax similarity scores were calculated (Methods), which can account for differences in syllable frequencies, and novel or omitted syllables. Comparison of Session A to Session B yielded syntactical similarity scores ~1.0, indicating a near perfect match between the two syntaxes (Fig. 1e, ‘unmodified’). In contrast, when 40 novel Bengalese finch syllables were randomly inserted into the Session B syntax (Fig. 1e, ‘modified’), the scores that penalize for the addition of novel syllable types dropped to ~0.75. Acoustic features were compared between clusters generated for Sessions A – B. Mean pitch and Weiner entropy, the latter being an acoustic measurement of syllable “noisiness,” were similar (p > 0.05, resampling independent mean differences) (Fig. 1f).

As a second method of validation, we compared results from VoICE with those derived from hand counts and sorting through visual inspection by an experienced birdsong analyst (Supplementary Note 2). Both analyses returned a similar number of syllable types (human: n = 8; VoICE: n = 7). The human observer characterized a small number (3/1105; 0.6%) of syllables as a distinct syllable type that the computer (and a second human observer) did not find as categorically ‘different enough’ to result in a separate syllable type, which demonstrates the influence of human bias on sound categorization.

### Quantification of deafening-induced song deterioration

**Figure 2:**
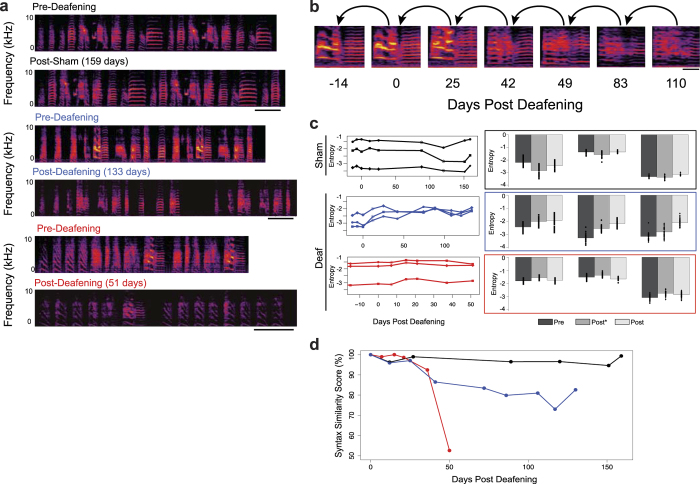
VoICE detects deafening-induced alterations in song phonology and syntax. (**a**) Spectrograms reveal song deterioration in deafened, but not sham-deafened, birds. (**b**) Syllables are assigned in a temporally-reversed serial manner to account for ongoing changes in syllable structure. (**c**) Syllable entropy, a measure of spectral ‘noise’, increases in a majority of syllables after deafening. Asterisks denote statistically significant changes from before surgery (left). Bar plots represent Pre (Day 0) vs. Post* (the first day statistically significantly different from ‘Pre’) vs. Post (the last analyzed day) recordings. Each symbol and line (left) and its corresponding pair of bars (right) represent a syllable cluster (right). (One-way resampling ANOVA, multiple comparisons post-hoc Bonferroni corrected p-value < 0.05) (**d**) Syntax similarity to pre-surgery decreases following deafening. (Black = sham; blue, red = deaf, * = p < 0.05 resampling independent mean differences. Scale bars = 250 msec in **a** and **b**.)

Like humans, zebra finches require auditory feedback to maintain mature vocalizations, and the degradation of zebra finch song structure and syntax in the absence of hearing is well characterized^17^,^18^. To demonstrate the utility of VoICE in tracking changes to vocalizations, two adult zebra finches (>120d) were deafened. Song deterioration and syntax impairment were evaluated over a 4-month time frame. Representative spectrograms illustrate the stereotypy of mature zebra finch song (Fig. 2a, pre-) and the variability in the time course of deafening-induced song changes (Fig. 2a, post-). Initial clusters were assembled from a pre-deafening singing epoch (Fig. 1a, top). Syllables from the first analyzed time point following deafening were then assigned to the pre-deafening clusters. For each subsequent time point, the first ~300 syllables from each day were assigned using the most recently clustered session (Fig. 2b). As syllables degraded, the global similarity floor was manually lowered to 35 to enable continual assignment, reduce tiebreaking, and prevent novel syllable classification. After all time points were clustered, Wiener entropy (Fig. 2c), and syntax similarity (Fig. 2d) were examined (for additional acoustic measures, see Fig. S2). As expected, syllable structure and syntax from a control bird (sham-deafened) were relatively unchanged throughout the recordings. In similarly-aged deafened birds, statistically significant changes to syllables were observed within 20 days (one-way resampling ANOVA, multiple comparisons post-hoc Bonferroni corrected p-value < 0.05). In comparison, changes to the syllables of the sham-deafened bird were smaller, in a different direction, and occurred after ~80 days, possibly reflecting ongoing behavioral precision with aging. The songs of the two deafened birds deteriorated in different domains – one had significant decreases in the entropy of his syllables consistent with syllable degradation (Fig. 2d, blue), whereas the other bird showed substantial decay in syntax (Fig. 2d, red), but only minor phonological changes. Both phenomena have been previously observed following deafening in this species, supporting the ability of VoICE in capturing key facets of birdsong^17^,^18^,^19^,^20^,^21^.

### Validation in determining mouse ultrasonic vocal repertoires

**Figure 3:**
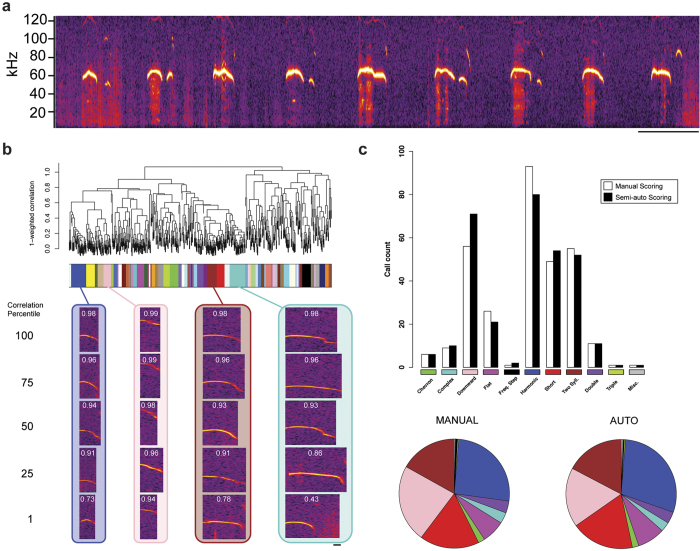
Validation of USV technique and comparison to manual classification standard. (**a**) Exemplar USVs from a mouse on the C57BL/6J background at P7. (Scale bar = 200 msec.) (**b**) A dendrogram generated following spectral similarity scoring of USVs where calls are represented as leaves and branch points indicate the difference in weighted correlation between leaves. Beneath the branches, clusters automatically determined by the tree-trimming algorithm are denoted by unique color bands and illustrated by representatives at descending percentiles following correlation of each cluster member to the cluster eigencall. The Pearson’s rho for the correlation between each syllable and the eigencall are displayed in white. (**c**) Bar plots indicate the count of each call type when the classification is performed manually (white) or using VoICE (black). Pie charts, right, illustrate the percentage distribution of each call type for the same animal’s repertoire as determined by manual sorting or using VoICE. (Scale bar = 10 msec.)

To validate VoICE in the analysis of USVs, a 5-minute recording session from a C57BL/6J mouse pup (P7) was examined using manual classification of calls, the current standard, and by using VoICE. In rodents, isolation-induced USVs are retrieval calls emitted by pups when separated from their mother, representing an infant-mother vocal communicative behavior thought to be relevant to autism spectrum disorder (ASD)^22^,^23^. Recordings revealed narrowband vocalizations in the ultrasonic range (~40–120 kHz; Fig. 3a). During manual scoring, a spectrogram of each call was generated in MATLAB, then assigned to one of 10 canonical call types^12^ or to three miscellaneous categories (Methods). The same set of calls was then analyzed using VoICE. The dendrogram created after acoustic similarity scoring (Fig. S3) and clustering was trimmed, then clusters that displayed an above-threshold level of homogeneity were described by calculation of an “eigencall” (Methods). All calls within each of these clusters were then correlated to their respective cluster eigencall and the single call with the highest correlation was selected as a representative of the entire cluster. Clusters were then classified by inspection of only these representatives. The clusters classified by a single representative call displayed a high level of within-cluster acoustic similarity (Fig. 3b). Syllables from clusters with a sub-threshold level of homogeneity were classified individually.

VoICE almost exactly duplicated the call type distribution achieved by hand sorting, indicating that it can replicate the result achieved by the current analytical standard (Fig. 3c, p = 0.96, resampling paired differences, Supplementary Note 2). The small non-significant differences observed between manual and semi-automatic classification methods mainly resided in more downward and fewer harmonic call assignments.

### Ultrasonic repertoire in *Cntnap2* KO mice

**Figure 4:**
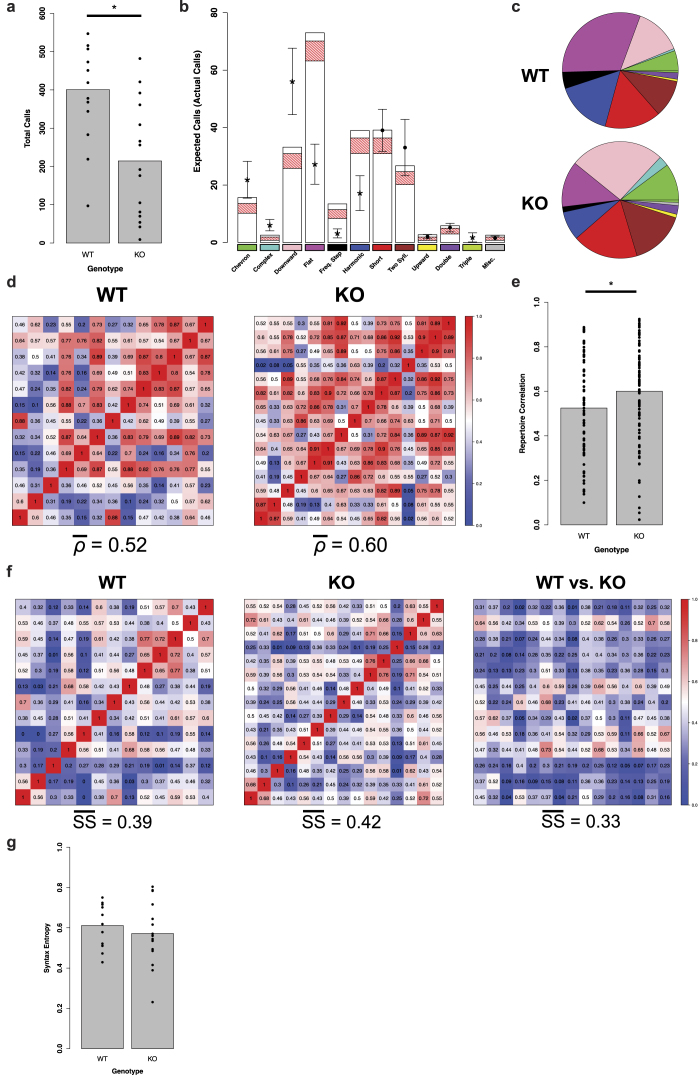
Deletion of *Cntnap2* results in altered vocal phenotype. (**a**) Mouse pups lacking *Cntnap2* (n = 15) do not call as much as WT littermates (n=13) (* = p < 0.05, resampling independent mean differences) (**b**) Expected counts of each call type (bars) generated from resampled WT data and 95% confidence intervals (red cross-hatch) reveal significant differences when compared to actual KO call counts, represented by overlaid points. Average counts of actual KO calls are represented as asterisks where p < 0.05. Error bars denote ±s.e.m. (**c**) Pie charts display the distribution of each call type in WT and KO animals. (Color scheme denoted beneath bars in **b**) (**d**) Heatmaps denote the correlation of repertoire within each genotype. KO animals show an intragenotype correlation greater than that of WT. Rows and columns represent animals, and indices are repertoire correlations between them. (**e)** Repertoire correlation is significantly greater within the KO genotype. (**f**) Heatmaps of the within- and across-genotype weighted unpenalized syntactical similarity scores show no within-genotype difference in syntax similarity. Rows and columns represent animals, and indices are syntax similarity scores between them. (**g**) Syntax entropy scores (a measure of call transition variability) within each genotype are similar.

The *Cntnap2* knockout mouse has been validated as one of the few mouse models of ASD with construct, face, and predictive validity^15^, making it useful for study of ASD pathophysiology. *Cntnap2* knockout pups emit a reduced number of USVs when separated from the dam^15^, however it is not known if this reduction is associated with abnormal spectral emission patterns. Other genetic mouse models of ASD have shown altered vocal repertoire^12^, allowing us to hypothesize that VoICE will detect a similar finding in the *Cntnap2* knockout (KO) mouse. Therefore, to demonstrate proof of concept, we analyzed recordings of vocalizations from *Cntnap2* KO and wild-type (WT) littermates, obtained from heterozygous crossings. At postnatal day 7 (P7), calls were recorded for 5 minutes and then processed using VoICE (Methods). The reduced call number previously reported in KOs^15^, was replicated here in a new cohort of animals (Fig. 4a).

Beyond call numbers, using VoICE to conduct a comprehensive analysis of the vocal repertoire in these mouse pups allowed us to observe the hypothesized differences between the genotypes for multiple call types. Relative to WT, a smaller amount of the *Cntnap2* KO vocal repertoire was devoted to flat, frequency step, and harmonic calls and a greater amount to chevron, complex, downward, and triple calls (p < 0.05, See “Statistical representation of ultrasonic vocal repertoire difference” in Methods, Fig. 4b,4c). These differences replicated trends observed in a smaller pilot cohort of pups of the same genotypes recorded in 2008 (data not shown), speaking to the robustness of both the phenotype and the analytic method. By using VoICE, we were able to assess the similarity of vocal repertoire across animals by computing the Pearson correlation between raw call counts for each animal within the two genotypes. The within-KO correlation (*ρ* = 0.60) was greater than that of the within-WT (*ρ* = 0.52), suggesting a restricted repertoire across all KO mice (Fig. 4d,4e, p = 0.0004, resampling independent mean differences).

Since VoICE results in classification of all vocalizations to a canonical call type, we were able to apply the same syntax analysis metrics used in the study of bird songs to the mouse pup calls. To do so, we quantified the average weighted unpenalized syntactical similarity between all pups within each genotype, and found similar within-genotype syntactical relationships (KO = 0.42, WT = 0.39, Fig. 4f), indicating that genotype has no effect on how similarly the animals order their calls. When compared across genotype, average syntactical similarity decreased (WT vs. KO = 0.33), an expected result given the difference in frequency of each call type between genotypes. Finally, we calculated syntax entropy scores, which describe the level of sequence variability with an animal’s vocal repertoire (Methods). Low syntax entropy scores indicate that vocal sequence is stereotyped (e.g. the occurrence of one call type very frequently precedes, or predicts, the occurrence of another call type). Syntax entropy scores did not differ between genotypes, indicating that genotype has no effect on how well the occurrence of one call type predicts the next (p = 0.41, resampling independent mean differences, Fig. 4g).

## Discussion

**Figure 5:**
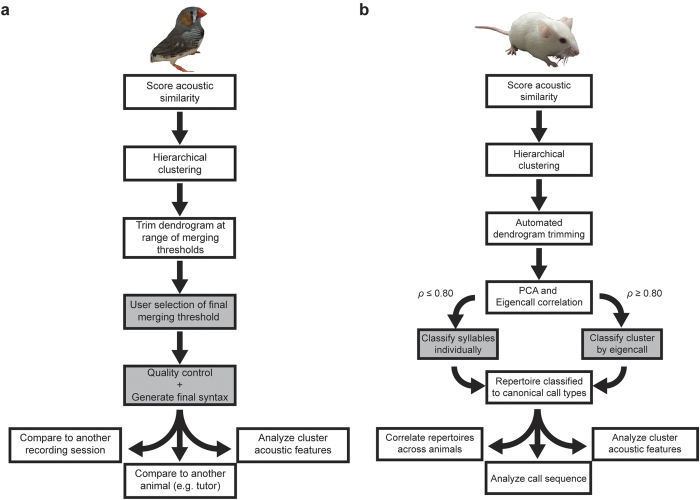
Summary of procedures. Flow charts describe the analytical pipeline for (**a**) zebra finch and (**b**) mouse USV analyses. Steps at which user input occurs are shaded in gray. (Animal photographs by NFD.)

We here present a new methodology to empirically derive distinct vocal repertoires in avian and rodent species in a streamlined and unbiased manner (Fig. 5). This is achieved by scoring acoustic similarity between individual song syllables or USVs and algorithmic trimming of a hierarchical cluster tree. We also provide a method for quantifying the syntactical similarity between bird songs and mouse USVs to assess the impact of experimental manipulation (e.g. auditory deprivation, gene knockout) on vocal behavior. These algorithms are not confined to zebra finch and mouse vocalizations, and can, in theory, be used to group VEs from any animal, so long as spectral co-similarity relationships can be appropriately quantified.

Our initial goal was to develop a method for grouping similar vocalizations together in the absence of input from the user, in order to generate a vocal syntax in an unbiased fashion. While an algorithm that intuitively trims dendrograms in an automated fashion should prove useful for accomplishing such a goal, user-defined thresholds that influence the number of clusters pruned from the tree still exist, prompting the need for empirical derivation of the “correct” number of syllable types in the animal’s repertoire. In the analysis of finch songs, our novel clustering approach provides the investigator with the appropriate information to make an accurate and reproducible decision in deriving vocal repertoire extent without actually viewing the syllables as they are grouped, thus maintaining a high degree of impartiality. Upon completion of clustering, should the experimenter deem his or her chosen merging threshold to be incorrect (e.g. by viewing the cluster spectrograms and determining that clusters were inappropriately split or joined), the selection of a new merging threshold does not implicitly dictate the merging or splitting of a given cluster, but instead allows a greater or lesser tolerance for variability within clusters (Methods, Supplementary Note 1).

In contrast to the highly stereotyped vocalizations of adult finches that are shaped through development and actively maintained in adulthood in the presence of auditory feedback, mouse pup retrieval calls are considerably more variable on a rendition-to-rendition basis. This variability makes the subjective derivation of call “sameness” exceedingly difficult. While VoICE still operates within the canonical call categorization system, by calculating acoustic similarity relationships and clustering syllables before their classification, the possibility of determining call subtypes within the canonical call classifications becomes possible, reducing the amount of within-type variability when quantifying acoustic properties. For example, the calls in each of the algorithmically defined clusters displayed in Fig. 3b all received a “downward” classification despite the obvious differences in frequency range, frequency contour, and duration across the clusters. Further, VoICE increases throughput by classifying entire clusters based on a single call mathematically determined to represent the greatest proportion of variance within the cluster. Accepting a greater level of variability when defining cluster cohesion, a determination that can be made based on experimental needs, can further increase the level of throughput.

Due to the algorithmic and mostly unsupervised grouping of VEs presented here, the quality of clustering is highly dependent on the quality of the input data and the measurement of spectral co-similarity scores. Should improvements be made in the scoring of similarity between vocalizations, the already high quality output of VoICE should increase. The extraction of songbird syllables and mouse calls is subject to user-defined segmentation parameters that both positively and negatively influence the spectral scoring of each sound. The start/stop boundaries of individual song syllables are manually determined in order to provide discrete syllable .WAV files as input to SAP’s “Similarity Batch.” For USV analysis, calls are extracted using a user-defined amplitude threshold, which can result in slightly truncated or elongated sounds. Additionally, sounds separated by less than 10 msec are grouped as a single call (which may result in “double” or “triple” categorization), because it is unknown what silence duration reliably distinguishes two distinct calls.

In birdsong analysis, the application of VoICE is not limited to the assessment of deafening-induced song deterioration. Assignment of syllables from a pupil bird to its tutor could separately quantify learning in the phonological and syntactical domains. Other potential experiments include clustering song syllables during a control condition (e.g. before injection of a drug or virus) and then comparing subsequent recording sessions to control song clusters to quantify changes in syntax and phonology as a result of the treatment.

ASD, a pervasive developmental disorder, is multigenic in origin. One core endophenotype is a deficit in speech and language skills, which impairs social communication and well-being^24^. Characterizing the vocal behavior of animal models will be critical in evaluating the role of autism-susceptibility and other (e.g. FOXP2) genes implicated in speech and language learning in an ongoing effort to test therapeutics for social communication disorders. Implementing analysis techniques that have benefitted the birdsong field may prove invaluable in the assessment of USVs in genetically modified mice. Here we used VoICE to reveal an altered vocal repertoire in mice lacking the autism susceptibility gene, *Cntnap2*. Though songbirds are excellent models for vocal learning and provide insight into the neural underpinnings of speech and language, it is currently more difficult to assess the underlying molecular determinants due to the challenge of generating and maintaining transgenic lines of birds^25^. Rodents are genetically tractable laboratory models, but the lack of standardization of USV analysis limits their efficacy in assessing genetic components related to vocalization. Our method provides an easy-to-use framework that can unify the analysis of both innate and learned vocal signals to provide insight into the genetic and physiological mechanisms that comprise vocal communication.

## Methods

### Software

All scripts and a manual containing step-by-step instructions for installation and conducting analyses are available at https://www.ibp.ucla.edu/research/white/CODE.html.

### Subjects

#### Finch

Three adult (>125 days) zebra finches (*Taeniopygia guttata)* were removed from our breeding colony (13:11 hour light/dark cycle). All animal husbandry and experimental procedures were in accordance with NIH guidelines for experiments involving vertebrate animals and approved by the University of California, Los Angeles Institutional Animal Care and Use Committee. Birds were fed seed and calcium-enriched (Calciboost, The Birdcare Company, Gloucestershire, UK) water ad libitum, provided with weekly nutritional and environmental supplements (hard-boiled chicken egg, fresh carrots and komatsuma, millet sprays, bathing water).

#### Mouse

All animal husbandry and experimental procedures were in accordance with NIH guidelines for experiments involving vertebrate animals and approved by the University of California, Los Angeles Institutional Animal Care and Use Committee. Pups (P7) were removed from the dam and placed in individual soundproof chambers equipped to record ultrasonic vocalizations for 5 minutes using an ultrasonic microphone with a flat frequency response up to 150 kHz and a working frequency response range of 10–180 kHz (CM16, Avisoft, Germany). To avoid any potential confounding effects due to temperature, the room was maintained at 21 °C. After recording, pups were tattooed and tail tissue was obtained to perform genotyping before returning them to the dam. Recordings were performed in the UCLA behavioral testing core. Mice were kept in 12 hr light/12 hr dark cycle and had ad-lib access to food and water. 

### Finch deafening surgeries

Following 2-3 weeks of baseline recording, two finches were deafened by bilateral removal of the cochlea as described by Konishi^26^. Briefly, birds were anesthetized with inhalant isoflurane and secured on a rotary table. Under a dissection microscope (OPMI pico, Carl Zeiss Meditec, Inc., Dublin, CA), a small area of skin as well as the tympanic membrane overlaying the middle ear cavity was removed using iridectomy scissors, followed by the removal of the columella, allowing visualization of the cochlea. A small hook made of tungsten fiber was used to extract the cochlea. Removal of an unbroken cochlea indicated the initial success of the surgery, which was later confirmed by deterioration of song. One additional bird underwent sham operations to control for any potential effects of the surgical procedure itself. Sham operations consisted of the anesthetic protocol and skin removal as the deafened birds, but without damage to the tympanic membrane or removal of the columella or cochlea. Following the procedure, Neosporin was applied to each ear and the animal was monitored in its recording chamber to ensure that the birds did not show life-threatening vestibular damage.

### Recording and sound preprocessing

#### Finch

Songs were recorded when birds were singly housed in sound attenuation chambers (Acoustic Systems; Austin, TX). Songs were recorded biweekly for approximately 7 months using Sound Analysis Pro^8^ (v. 2011.107) recorder using Countryman EMW Omindirectional Lavalier microphones (Countryman Associates; Menlo Park, CA) attached to the chamber ceiling at a fixed location. Sounds were digitized using a PreSonus Firepod at a sampling rate of 44.1 kHz and 16 bit depth. For each day of analysis, the first ~300 syllables were semi-automatically extracted using the SAP “Explore & Score” module. Raw sound recordings were opened in SAP’s “Explore & Score” module (Fig. 1), then a segmentation threshold was applied to delineate syllable boundaries. Syllable tables were then built by highlighting syllables sequentially and exporting their records to an SQL table, where individual rows represent syllables in the sequence in which they were sung, adjusting segmentation parameters as needed to ensure optimal syllable start and end boundaries. The syllable tables were then imported to R, where the syllable onset and duration data were used to automatically clip each syllable from the raw recording to its own WAV file.

#### Mouse

Calls were recorded over a single 5-minute recording session for each mouse at postnatal day 7 (P7) between 9:00 AM and 10:00 AM during their light cycle. To avoid any potential confounding effects due to temperature, the room was maintained at 21 °C. To isolate individual calls from the raw recordings, we used a MATLAB script that calculated the amplitude envelope in each file and clipped into individual WAV files all sounds that passed the threshold for longer than 2 msec. Calls were considered complete after the clip fell below threshold for 10 msec. Finally, all sounds longer than 150 msec were considered to be noise and discarded from the analysis. To provide the highest quality input data to our clustering workflow, we then manually inspected WAV files and discarded those that contained only noise.

### Similarity scoring

#### Finch

Similarity scores between syllables were calculated using a MATLAB algorithm adapted from the C++ code for the SAP similarity batch function. Briefly, syllables were quantified millisecond-by-millisecond based on four features: Weiner entropy, frequency, FM, and pitch goodness (a measure of sound periodicity). These features were calculated in 9.3 ms bins with sliding 1-ms steps with a subprogram of SAP, ported to MATLAB by S. Saar. Upon completion of the similarity batch, global similarity (GS) scores (Fig. 1) for each syllable-syllable comparison were calculated by dividing the product of similarity, accuracy, and sequential match (“temporal overlap”) by 10,000, a metric employed previously to describe the overall likeness between song types following deafening^17^.

#### Mouse

Our original intent was to score acoustic similarity between USVs in a fashion similar to bird song syllables. Due to the great difference in bandwidth between bird song syllables and mouse USVs, this metric indicated a high level of similarity between all calls. Therefore, we designed a different metric focused on describing relationships between the frequency contours of the mostly narrowband calls. All recordings were sampled at 250 kHz, then filtered so that only spectral data between 40 and 120 kHz was considered. Each call was transformed to a spectral derivative in MATLAB. Calls were then binned into windows of 44 samples each and the average pitch across all samples was calculated for each window. The median pitch across every 5 consecutive windows was then calculated and used, therefore allowing us to describe the overall pitch for every ~0.9 msec of each call.

Once frequency contours were calculated, the pitch scores were then compared between all calls in a pairwise fashion. First, the Pearson correlation of raw pitch was calculated for all overlapping windows between each pair of calls in order to describe the similarity of frequency contours. The correlation of raw pitch is independent of time and the frequency range in which the two calls reside, therefore scoring similarity simply by correlation in pitch could lead to similar frequency contours at vastly different frequency ranges appearing highly alike. Therefore, we took additional measures to weight pitch correlation to more accurately describe the relationships between calls. First, the absolute difference in pitch at each overlapping ~0.9 msec window was calculated and averaged across all windows, divided by the maximum possible difference in pitch (or 80 kHz, as dictated by the frequency range over which we analyzed spectral data), then subtracted from 1. Calls occupying a similar frequency range will have very low absolute differences in pitch, leading to a pitch difference score close to 1. Finally, to account for differences in duration between pairs of calls, the temporal overlap was calculated. The product of these three measures, pitch correlation, scaled pitch difference, and temporal overlap resides on a -1 to 1 scale and was used as the input to the hierarchical clustering function (Fig. S3).

#### Both species

Before implementation of the hierarchical clustering function, acoustic similarity scores were transformed to an M x M (M = number of vocalizations) similarity matrix. For finch syllables, the Euclidean distance between all syllable-syllable pairs in the GS matrix were then calculated, generating an M x M distance matrix, which is used as the input to an average linkage hierarchical clustering algorithm. For mice, all values in the weighted correlation matrix are subtracted from 1 and used as the dissimilarity input to the clustering algorithm.

### Hierarchical clustering

#### Both species

The dissimilarity matrix generated in the previous step is used as input to the flashClust function^27^ in the weighted gene co-expression network analysis (WGCNA;^28^) package in R (http://r-project.org). Syllables are clustered by average linkage hierarchical clustering to generate a dendrogram. Hierarchical clustering is desirable for this type of analysis, as it does not require dictation of a number of call types as other clustering methodologies do. The hierarchical cluster tree is trimmed using the Dynamic Tree Cut R package ‘Dynamic Hybrid’ tree cut function^14^. As opposed to dictating a static tree cut height and generating clusters based on which branches remain together following trimming at this user-dictated height, this algorithm climbs bottom-up through hierarchical trees and derives clusters in an automated fashion based on user-dictated parameters. Despite its mostly automated function, the tree-cutting algorithm is tunable to an extent that can influence the number of clusters that are gleaned from the hierarchical tree. To be as rigorous as possible, we dictated that the minimum cluster size to be a single vocalization and set the deepSplit parameter to its highest level of sensitivity in cluster detection. We offer the user the ability to tune these parameters to suit their own needs.

### Eigensyllable/eigencall calculation

#### Both species

Following tree-trimming, each multi-vocalization cluster is given a unique color name and is described by calculating a cluster “eigensyllable” or “eigencall”, defined as the first principal component of the acoustic similarity scores within the cluster as determined by singular value decomposition^28^. The eigensyllable or eigencall can be considered as a single representative of the acoustic properties of all the vocalizations within a cluster.

### Iterative cluster merging

#### Finch

Since it is possible to ascertain a “correct” number of distinct syllable types in zebra finch song, small clusters initially derived through the divisive trimming of the dendrogram are merged together. The Pearson’s product-moment correlation between each cluster eigensyllable is calculated and clusters whose eigensyllables correlate above a user-provided Pearson’s rho threshold are merged together. This process then repeats until no cluster eigensyllables correlate above the given threshold. Since, ultimately, the number of cluster merges performed by the automated tree-trimming algorithm determines the number of clusters and, therefore, the number of syllable types in the animal’s repertoire, we sought to develop an iterative procedure to empirically derive the ideal merge threshold.

The iterative procedure is as follows: the hierarchical tree is created, then the automated tree-trimming algorithm is applied to the tree using the most divisive parameters possible, creating numerous small clusters. Each small cluster is represented by an eigensyllable. The iterative procedure then begins, where clusters whose eigensyllables correlate at or above 0.99 are merged. The rho is then decreased to 0.98 and the process repeats. This process repeats at each rho until reaching 0. At each step, the number of clusters and the average intracluster global similarity score (IGS) are calculated. As the merging threshold approaches zero, the number of clusters and the IGS for each merged pair of clusters decreases. One would expect the ideal cluster number (n) to remain stable over a large range of merging thresholds, as this would indicate that each cluster is sufficiently dissimilar to every other cluster that it will not be merged (unless the threshold for merging becomes so low as to allow for enough intracluster variability to override concrete differences in syllable spectral properties). When the merging threshold decreases to a point where all clusters that do not display a level of anticorrelation are joined together, a floor for the possible number of syllable types is reached. Upon completion of the iterative cluster merging, the IGS and the number of syllables in each cluster at each cluster n that remained stable over multiple merging steps are returned to the user. The user then selects the appropriate cluster n by considering the degree of IGS and the expected number of syllable types in the animal’s repertoire.

Once an appropriate merging threshold has been selected, a list is returned to the user containing: (1) the data, in the form of an M x M (m = number of syllables) distance matrix, worked on by the clustering algorithm, (2) the song syntax before applying any merging techniques, (3) the song syntax at the derived merging threshold, (4) the eigensyllables, and (5) the proportion of variance in each cluster explained by its eigensyllable. WAV files of every syllable within each cluster are then generated and presented to the user for purposes of error checking. Manual error correction is accomplished by changing the cluster ID for each syllable within the merged song syntax component of the list generated during clustering. Once clusters are finalized, the syntax is returned and the acoustic data are sorted into tables for each cluster.

### USV cluster quality control and eigencall-based classification

Unlike the crystallized and highly stereotyped vocalizations of adult finches, a high degree of call-to-call variability in the mouse ultrasonic vocal repertoire exists. Applying a merging strategy similar to the one implemented for finches resulted in few cluster ns that stayed stable over multiple merging thresholds, indicating that the number of distinct call types is highly sensitive to the amount of variability allowed within the cluster, making it impossible to ascertain the “correct” number of syllable types. This drove us to implement a cluster quality control step before dictating all calls within a cluster as “the same.” For each cluster defined by the tree-cutting algorithm, the Pearson correlation between all calls within the cluster and the cluster eigencall are calculated. Clusters whose average Pearson correlation is below a user-defined threshold (for all data described in this manuscript, a correlation threshold of ≥ 0.8 was used) are considered insufficiently cohesive and syllables within are classified manually. Clusters that do show sufficient correlation are then represented by the single syllable within them that displays the highest Pearson correlation to the cluster eigencall.

Following quality control, individual calls from dissolved clusters and/or single-call representatives of individual clusters were classified into the canonical types as described by Scattoni et al.^12^. In some cases, our method for automatically clipping calls from the raw recordings resulted in multiple calls being clipped into one WAV file. We therefore added categories called “double” and “triple” to reflect when this occurred. Finally, we observed a small but not unsubstantial number of calls that did not display the characteristics of any of the canonical calls. Instead of forcing these into a category in which they do not fit, we added a “miscellaneous” category to which we assigned these syllables.

### Assignment of syllables to established clusters

The preceding clustering step generates syllable clusters for a single animal within one recording session. In order to relate a second recording session to clusters created in an initial session, a similarity batch is first performed between all syllables in Session B and a subset of syllables in Session A (sorted by cluster). GS is calculated for all comparisons. A subset of syllables is selected from each Session A cluster to decrease computation time in the similarity batch. Should every member of each Session A cluster be included, nearly redundant similarity comparisons between a Session B syllable and highly alike Session A syllables would occur, increasing computation time without providing useful similarity data. The syllable subset from each session one cluster is determined by calculating the correlation between all syllables in each cluster to their respective cluster eigensyllable. The correlations are then ranked and the top 10% are used as representatives of the cluster, as these syllables statistically represent the greatest proportion of variance within the cluster. In our initial testing, using the top 10% resulted in nearly identical syllable assignment results as using the entirety of each cluster with a ~90% savings in processing time, though the user ultimately can select the percentage of the cluster to use. To prevent inappropriate syllable assignment to a cluster in the case of an improvised syllable during the second recording session, a GS floor is dictated by the user, below which a syllable will not be assigned to a cluster. The assignment of the GS floor is at the discretion of the experimenter, with a lower floor used in cases where acoustic similarity between recording sessions is not expected to be high, such as in the case of assigning a juvenile pupil bird’s syllables to a tutor’s clusters or syllables obtained following a deafening procedure to clusters created from pre-deafening song.

Syllables from the second recording session are then considered one at a time in their relationships to the representatives of the established clusters. For all established clusters of which an unassigned syllable shares an above-threshold GS, a one-way ANOVA and pairwise comparisons post-hoc test are performed to determine whether the unassigned syllable shows a statistically significant relationship after Bonferroni p-value correction with one cluster above all others. In the case that a significant relationship is determined, the unassigned syllable is given the same cluster ID as the established cluster. In the case that no significant relationship is observed, yet multiple clusters existed above the GS threshold, the unassigned syllable is passed into a tiebreaking queue (see below). Lastly, in the occurrence of an unassigned syllable that does not show an above-threshold global similarity relationship with any established cluster, it is deemed a novel syllable and passed into a queue for later derivation of the number of novel syllable types present.

Once the first pass through syllable-to-cluster assignment has finished, a round of tiebreaking occurs. All syllables that were passed into the tiebreaking queue have met the following conditions: (1) showed an above-threshold GS with more than one established cluster and (2) did not show a statistically significant relationship with one cluster above all others. From this point, the user is allowed to view the spectrograms of the unassigned syllable and the syllable within each established cluster that displays the highest correlation with that cluster’s eigensyllable. The user then clicks a button to assign the syllable to any cluster or add it to the unassigned syllable queue.

Upon completion of the previous two cluster assignment steps, the remaining syllables that did not get passed into a cluster are considered in an effort to determine the number of syllable types present in the second recording session that are not present in the first (e.g. syllables sung by a pupil that are not in its tutor’s vocal repertoire). A similar procedure to the initial clustering step is performed, utilizing the GS scores between each unassigned syllable and each representative of each session one cluster. Unassigned syllables that show similar relationships to the established clusters are likely similar to one another. Using this logic, first, an M x N (M = number of unassigned syllables from session two, N = total number of cluster representatives from session one) GS matrix is created. This GS matrix is then transformed to an M x M correlation matrix by calculating the Pearson correlation between unassigned syllables. This correlation matrix is then transformed to a dissimilarity matrix by subtracting all correlations from one. The dissimilarity matrix is used as the input to the hierarchical clustering function, whose dendrogram output is, again, trimmed iteratively over a range of merging thresholds as described above.

Since no GS scores are calculated between session two syllables, the measure of cluster cohesiveness determined at each merging threshold is the proportion of the variance explained by the cluster eigensyllable, ranging from 0 to 1. As clusters are merged and greater variability is added to each cluster, the proportion of variance explained by each eigensyllable decreases. As with the initial clustering step, novel numbers of clusters that remain stable over multiple merging thresholds are returned along with the proportion of variance explained by each eigensyllable and the user must select the appropriate merging threshold. At this point, spectrograms are created and presented for validation. Upon arrival at an appropriate merging threshold, the second session syllable sequence is named by cluster assignment to generate a syntax string suitable for comparison with the first recording session.

### Quantification of syntactical similarity

Syntactical similarity between two recordings (e.g. one recording session vs. another of the same animal) was determined by creating separate transition probability matrices for each song based on cluster assignments. The transition probability between two syllables is calculated by summing the total number of transitions between a leading syllable type and a following syllable type, then dividing by the total number of transitions between the leading syllable type and all syllable types. This calculation is performed for all possible syllable-syllable pairs, including self-transitions.

Following construction of transition probability matrices, the transition behavior of the same syllable in both recording sessions is compared by calculating the Pearson correlation between corresponding rows. High correlation between rows indicates similar transition relationships for a given syllable in the two songs recordings being compared. To account for possible differences in frequency of syllable occurrence between the two recordings (e.g. the animal emits numerous renditions of vocalization ‘A’ in one recording session and very few in another), one minus the absolute difference in syllable type frequency (= the ratio of number of renditions of a given vocalization type to the total number of all vocalization types) is multiplied by the Pearson correlation for each corresponding row in the transition probability matrices. Finally, to obtain an overall score for syntactical similarity, the average Pearson correlation between corresponding rows of the transition probability tables, weighted by the difference in syllable type frequency, is calculated. It is worth noting, however, that correlation between individual rows can be informative to discover whether certain vocalization types’ transition behavior is more responsible for driving an overall change in syntactical similarity.

In the event where one recording contains at least one vocalization type that is not present in the other (e.g. a novel vocalization emerges following an experimental manipulation), the transition probability matrices are modified so that the unique vocalization type is represented as a row and column of zeroes in the transition probability matrix for the session the novel syllable type was not present in. Therefore, when correlations between rows in transition probability matrices are calculated, the unique syllable type results in a correlation of zero, providing a penalty when averaging all correlations. To quantify syllable transition similarity for only the syllable types that are present in both sessions, the averaged correlation only includes information for transitions between syllables found in both recording). In addition to the weighted scores, our method also returns the unweighted versions, which do not account for potential differences in syllable occurrence between recording sessions. The syntactical similarity metrics are summarized in Table 1.


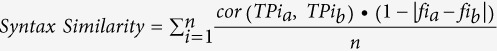


n = number of syllable types; TP = transition probability; a, b = recording sessions; f = syllable frequency

### Syntax entropy scores

Syntax entropy for both species is calculated by performing ‘string-based’ sequence analysis as in Miller *et al*., 2010.^29^

### Statistical representation of ultrasonic vocal repertoire difference

To statistically assess the genotype effect on vocal repertoire, we employed a resampling technique where the actual WT call distribution was used to generate an expected call distribution for the KO animals. The median count of each call type across all WT animals was calculated and used to generate a resampling pool. From this pool, the total number of calls made by the actual KO animals was drawn, with replacement. The count of each call type in this resampled distribution was then stored. This process was repeated 10,000 times, generating 10,000 counts of each call type expected should the gene knockout have no effect. The 95% confidence interval was calculated for the distribution of each call type. Finally, to determine statistical significance, the average count of each call type produced by the KO animals was compared to the resampled distribution. If the average count ± SEM fell outside the 95% confidence interval of the resampled data, a statistically significant over- or underrepresentation of that call type was said to result from the gene knockout.

### Resampling independent mean differences

A resampling version of the Student’s T-test is performed by, first, calculating the difference in mean value for the data as observed. To simulate the null hypothesis, individual data points for both groups compared are gathered into a resampling pool, which is drawn from randomly, of equivalent n to the observed groups, and with replacement to generate simulated representations of the actual data. The difference in mean for the simulated groups is calculated and stored. This process was repeated 10,000 times to generate a distribution of means arising from data simulated under the null hypothesis. The difference in mean for the actual data is then compared to this distribution. Exact p-values are calculated by quantifying the number of resampled means more and less extreme than the actual mean difference and its inverse, then dividing by the total number of resamplings.

### Resampling paired differences

A resampling version of the Paired T-test is performed by, first, calculating the difference between pairs of data, to generate a vector of differences. The average of these differences is then calculated. To simulate the null hypothesis, the signs for the differences are randomly inverted and a new mean is calculated. This process of randomly inverting the signs for the vector of differences and calculating the mean was repeated 10,000 times to generate a distribution of differences under the null hypothesis. The difference that was actually observed is then compared to this distribution. Exact p-values are calculated by quantifying the number of mean differences more and less extreme than the actual mean difference and its inverse, then dividing by the total number of resamplings.

## Additional Information

**How to cite this article**: Burkett, Z. D. *et al.* VoICE: A semi-automated pipeline for standardizing vocal analysis across models. *Sci. Rep.* **5**, 10237; doi: 10.1038/srep10237 (2015).

## Supplementary Material

Supplementary Information

## Figures and Tables

**Table 1:** Summary of syntax similarity scores and description of transition behavior quantified by each metric.

Unweighted	**Weighted**	**Unpenalized**	**Penalized**	**Description**
x		x		Ignores vocalization frequency and novelvocalizations between sessions
x			x	Ignores vocalization frequency, accounts fornovel vocalizations between sessions
	x	x		Accounts for vocalization frequency, ignoresnovel vocalizations between sessions
	x		x	Accounts for vocalization frequency andnovel vocalizations between sessions
