# Supplementary material for: VoICE: A semi-automated pipeline for standardizing vocal analysis across models
Source: Sci Rep. 2015 May 28;5:10237. doi: 10.1038/srep10237 (PMC4446892; doi:10.1038/srep10237)
Supplement: Supplementary Information [file srep10237-s1.pdf]

VoICE: A semi-automated pipeline for standardizing vocal analysis across models

SUPPLEMENTARY MATERIAL

Zachary D. Burkett*[1,2], Nancy F. Day[1], Olga Peñagarikano[3,4], Daniel H. Geschwind[3,4,5], & Stephanie A. White[1,2]

[1]Department of Integrative Biology & Physiology, University of California, Los Angeles, California 90095

[2]Interdepartmental Program in Molecular, Cellular, & Integrative Physiology, University of California, Los Angeles, California 90095

[3]Program in Neurogenetics, Department of Neurology, David Geffen School of Medicine, University of California, Los Angeles, California 90095

[4]Center for Autism Research & Treatment, Semel Institute for Neuroscience & Human Behavior, University of California, Los Angeles, California 90095

[5]Center for Neurobehavioral Genetics, Semel Institute for Neuroscience & Human Behavior, University of California, Los Angeles, California 90095

*Corresponding Author: zburkett@ucla.edu

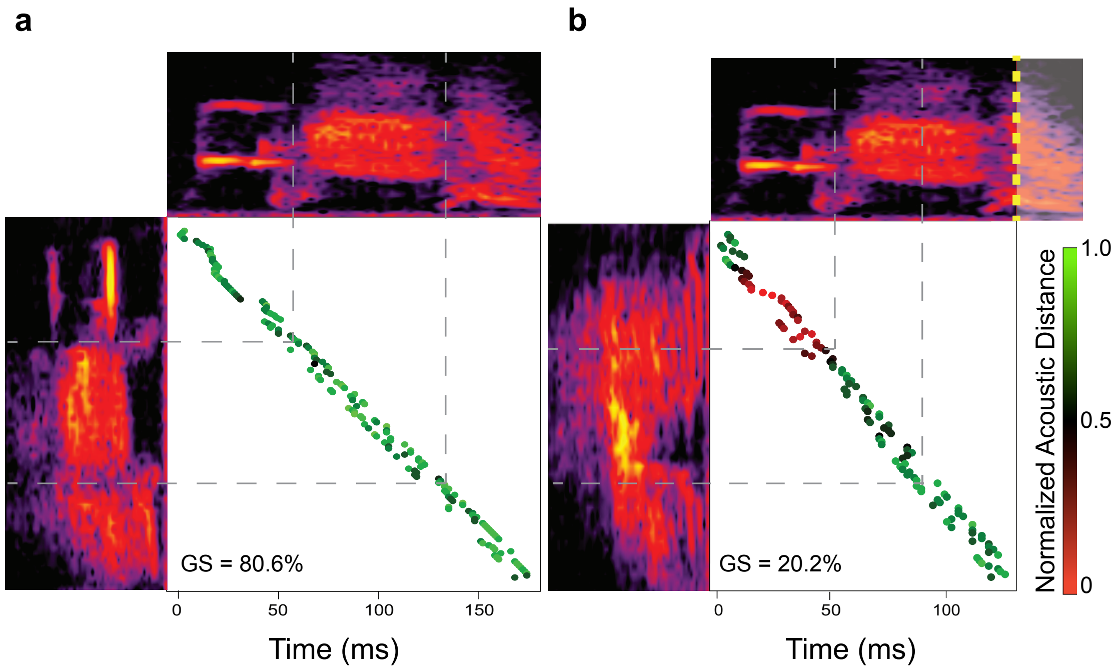

Figure S1 | Zebra finch acoustic similarity scoring. Similarity scores are determined by averaging the millisecond-by-millisecond Euclidean distance of four acoustic features: pitch, Wiener entropy, frequency modulation, and goodness of pitch. (**a**) Visually alike syllables are highly similar at each millisecond (green dots), and (**b**) distinct syllables are more dissimilar (red dots). The global similarity score (GS), which is partially determined by differences in syllable duration, for each pair of syllables is displayed in the lower left-hand corner of each plot. (Spectrogram frequency axis range 0 to 10 kHz.)

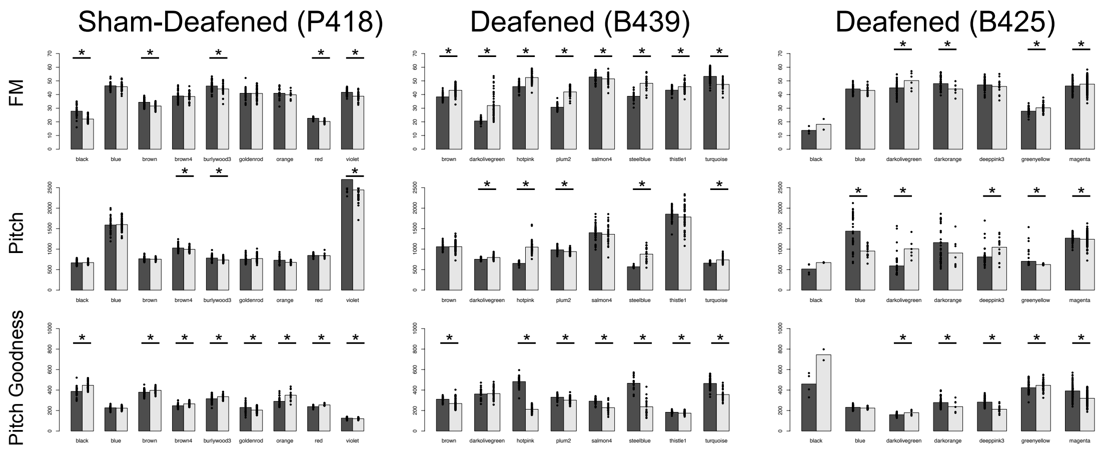

Figure S2 | Quantification of multiple acoustic features before and following deafening. The mean (**a**) frequency modulation (FM), (**b**) Pitch, and (**c**) Pitch Goodness for each cluster of a sham-deafened bird and two deafened birds reveals the consequences of auditory manipulation in each bird. Each dot represents a single syllable. (* = p<0.05, resampling independent mean differences.)

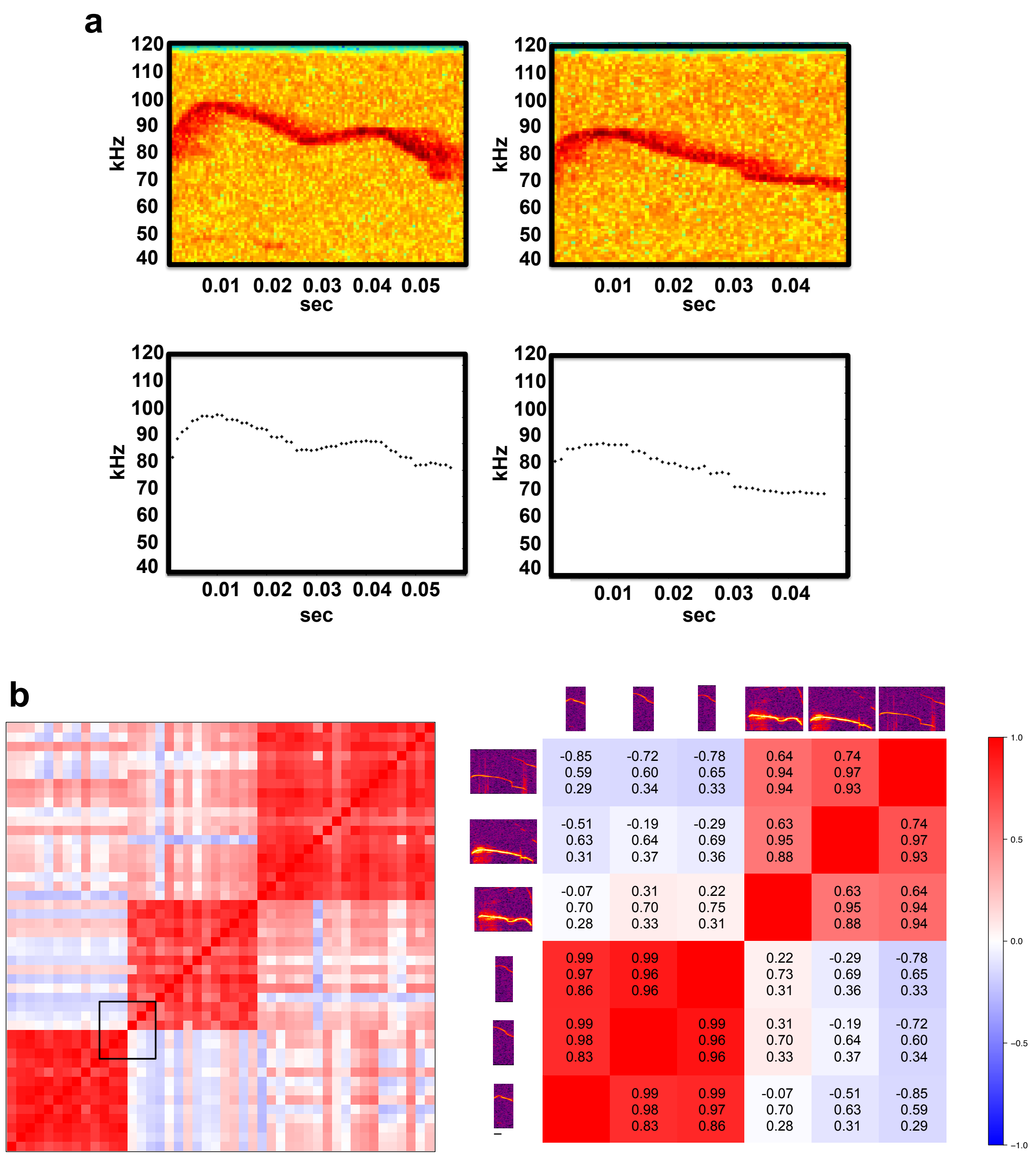

Figure S3 | USV similarity scoring. (**a**) Individual USV spectrograms (top) are transformed to frequency contours summarizing 0.9 ms windows before similarity scoring (bottom; see **Online Methods**). (**b**) Exemplar USV weighted correlation matrix used as input to hierarchical clustering algorithm represented as a heatmap (left) and inset, black square (right), illustrating actual syllables and their pitch correlation, pitch difference, and temporal overlap scores (top to bottom, respectively). Rows and columns in the heatmap represent calls from one animal's recording session. The indices represent the spectral similarity scores between each pair of calls. Three clusters automatically defined by the tree-trimming algorithm were used as exemplars. (Scale bar = 10 msec.)

Introduction to Supplementary Notes

The ideal system for clustering songbird syllables is an errorless and unbiased observer, which does not exist. Further, hand parsing of data is not feasible when considering thousands of syllables. Nevertheless, to evaluate VoICE, an hour's worth of song recordings from a zebra finch were manually clustered by an experimenter familiar with song analysis but blinded to the analytical goal, then the same set of recordings were passed through the VoICE pipeline by another experimenter familiar with the procedure.

The human observer found 1122 syllables in the hour of songs. The experimenter using VoICE found 1105 syllables in the same hour, with the minor discrepancy likely due to difference of opinion between experimenters as to the initiation and termination of song bout boundaries. Still, both experimenters largely considered the same song content in their respective analyses.

Supplementary Note 1: Selecting a Merging Threshold

After construction of the M x M distance matrix as outlined in Online Methods, a dendrogram was created and trimmed, resulting in the creation of 65 unique clusters (Figure 1A, 'unmerged'). The tree trimming procedure was then iteratively repeated and the merging threshold decreased from 1 to 0 by steps of 0.01 with each iteration. Upon completion of iterative tree trimming, the cluster numbers that remained stable over at least two merging thresholds were set aside for further analysis (Figure 1B).

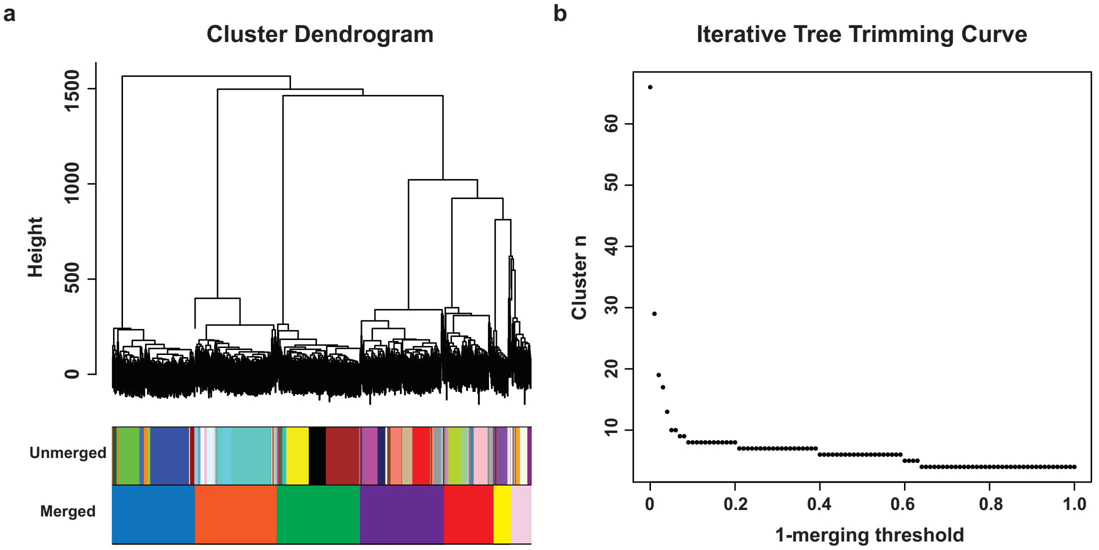

Figure 1 I Detailed clustering results. (**a**) A dendrogram generated for 1105 syllables recorded during one hour of singing. 'Unmerged' colors represent the most divisive trim by the automated tree-trimming algorithm. 'Merged' colors are groups following guided dendrogram trimming, consisting of cluster ns that remained stable over multiple merging thresholds in (**b**).

At each stable merging threshold, the user is then presented with the IGS for each cluster and the number of syllables present in that cluster. Ultimately, the user must determine the correct merging threshold by weighing the balance between the number and size of clusters, IGS, the number of merging thresholds over which the cluster n remained constant. Six unique cluster definitions were stable over multiple merging thresholds, narrowing the possible number of syllable types to a range between five and 10 (Table 1).

Table 1 | Number of clusters, number of syllables in each cluster (n_{syl}), and IGS at the first merging threshold that resulted in a stable cluster n at over at least two merging threshold changes.

Threshold	Cluster ID	n_{syl}	IGS
0.94	red	128	83.6
(n = 10)	orange	221	81.8
	green	221	86.0
	blue	221	82.6
	yellow	38	85.1
	pink	45	70.4
	purple	227	82.1
	cyan	3	85.7

	magenta	2	53.8
	tapioca	2	35.5
0.92	red	128	83.6
(n = 9)	orange	221	81.8
	green	221	86.0
	blue	221	82.6
	yellow	38	85.1
	pink	47	65.7
	purple	227	82.1
	cyan	3	85.7
	magenta	2	53.8
0.9	red	128	83.6
(n = 8)	orange	221	81.8
	green	221	86.0
	blue	221	82.6
	yellow	38	85.1
	pink	49	63.2
	purple	227	82.1
	cyan	3	85.7
0.79*	red	128	83.6
(n = 7)	orange	221	81.8
	green	221	86.0
	blue	221	82.6
	yellow	38	85.1
	pink	49	63.2
	purple	230	80.4
0.58	red	128	83.6
(n = 6)	orange	221	81.8
	green	221	86.0
	blue	221	82.6
	yellow	38	85.1
	pink	279	64.3
0.39	red	407	52.0
(n = 5)	orange	221	81.8
	green	221	86.0
	blue	221	82.6
	yellow	38	85.1
0.32	red	407	52.0
(n = 4)	orange	221	81.8
	green	259	69.6
	blue	221	82.6

Asterisk denotes the merging threshold chosen and illustrated in Figure 1 (‘merged’).

The merging thresholds close to 1 resulted in high cluster n, coincident with the existence of fewer, smaller clusters. The presence of multiple distinct syllable types with very few renditions each in an adult zebra finch’s song is unlikely, suggesting that utilizing a very high merging threshold is too strict and results in under-merging of clusters. Conversely, merging thresholds close to 0 resulted in a low cluster n with relatively low IGS due to increased heterogeneity within the

cluster. Based on these observations, merging thresholds at the extremes of the spectrum were removed from consideration (0.94, 0.92, 0.58, 0.39, 0.35).

Finally, merging thresholds of 0.9 (n=8 clusters) and 0.79 (n=7 clusters) were considered. When the threshold is lowered to 0.79, the cyan cluster (n=3 syllables, IGS=85.69) is merged into the purple (n=227 syllables, IGS=82.12) cluster. Following this merge, the purple intracluster identity decreases to 80.43 (Table 1), indicative of an average score of 93 for similarity, accuracy, and sequential match between all syllables in the cluster. When the hierarchical tree was trimmed using the 0.79 merging threshold, seven clusters were generated (Figure 1A, 'merged'). Manual error checking of clusters revealed that two syllables were placed in the incorrect cluster, resulting in an error rate of ~0.18%.

Supplementary Note 2: Comparison to Human Scoring

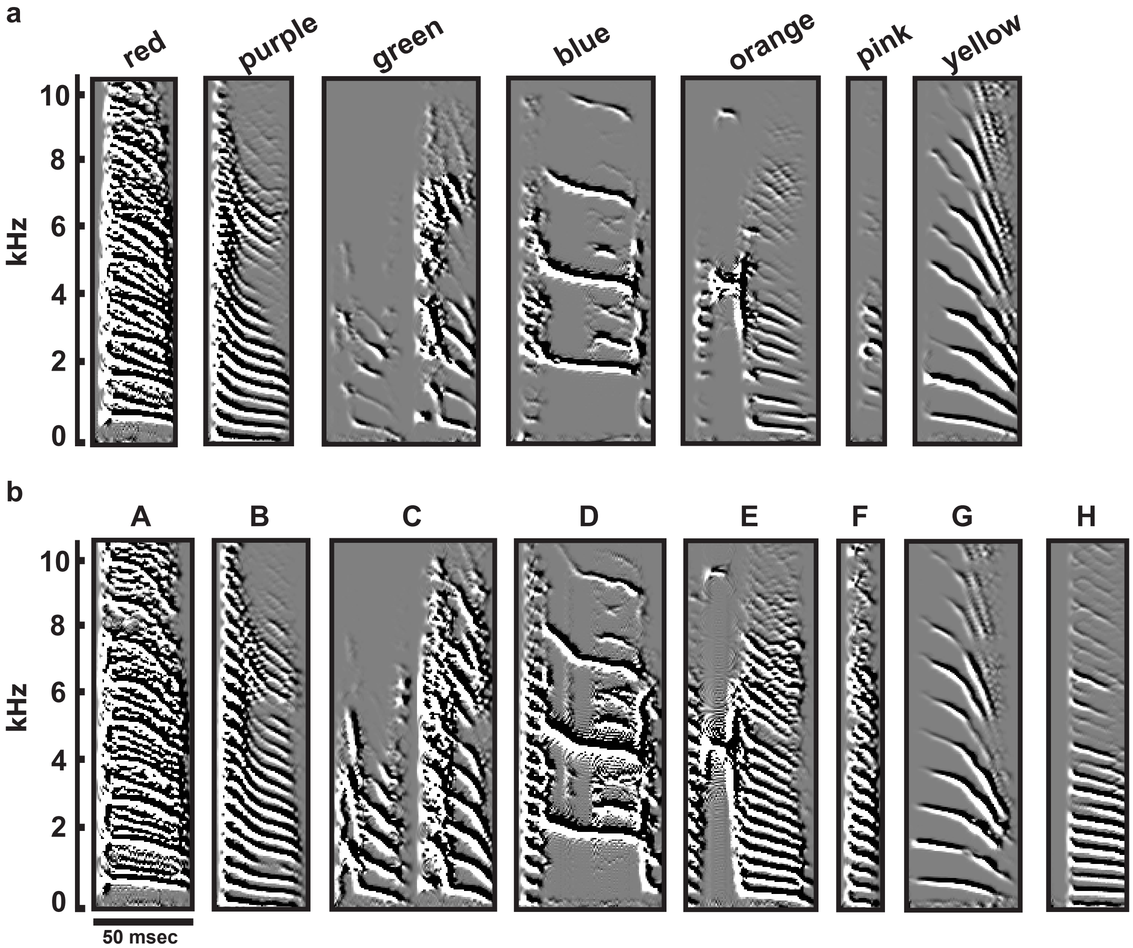

Figure 2 I Comparison of unique clusters determined by different methods. (**a**) Seven syllable clusters were determined by guided dendrogram trimming using VoICE. (**b**) Eight clusters were assessed by the experimenter scoring by hand.

A discrepancy occurred in the number of syllable types present as determined by the experimenter manually clustering the syllables (n = 8, Figure 2A) versus using the threshold determined by iterative trimming of the hierarchical tree (n = 7, Figure 2B). When sorted by hand, the syllable type determined by clustering, purple, was subdivided into syllables B (n=224 syllables) and H (n=7 syllables) (Figure 2). The merge in question eliminates the presence of a cluster containing only three syllables in a total of 1105. It is possible that an adult zebra finch could sing a distinct syllable type as ~0.6% of its song, but the more parsimonious interpretation is that syllable H, while somewhat dissimilar from syllable B, is still of the same "type." Indeed, syllables B and H are largely similar: both are of approximately the same pitch (median = 375.5 hz and 402 hz, respectively) and duration (median = 55.87 msec for both), though syllable B is slightly more frequency modulated (median = 40.5 vs. 22.15). Therefore, for the purpose of comparing syntax scores between the manual vs. semi-automated approach, all syllables scored as "H" were renamed to "B." When considering the syllables for the purposes of acoustic analyses, however, one can opt to deem syllable H as a subtype of B (e.g. B_i) and consider their acoustic properties separately.

To compare the two methods for quantifying song syntax, transition probability tables were created and these methods resulted in very similar scores, with the advantages of VoICE being faster in the processing of larger data sets and introducing less experimenter bias. There were marginal differences found between the two methods and transitions that were present in one analytical method that did not exist in the other were inspected more closely (Table 2).

Table 2 | Comparison of transition probabilities between VoICE (top) and manual scoring by an investigator (bottom).

	Lead	Following Syllable						
Method	Syllable	Red	Purple	Green	Blue	Orange	Pink	Yellow
VoICE	Red	5.5	94.5					
	Purple	3.9		96.1				
	Green				100.0			
	Blue					100.0		
	Orange	11.4	49.1				22.3	17.3
	Pink	100.0						
	Yellow	97.4	2.6*					
Manual	Red	5.4	94.6					
	Purple	3.0		97.0				
	Green				100.0			
	Blue					100.0		
	Orange	11.7	48.4				22.4	17.5
	Pink	100.0						
	Yellow	100.0						

Only one transition absent in the hand sorting of syllables was present when syllables were clustered using VoICE. This amounted to a single yellow to purple transition. This discrepancy was potentially attributable to one of two possibilities: an error resulting from the procedure or the clustering analysis including a syllable that was not deemed part of a song bout by the experimenter sorting syllables by hand. The latter proved to be true as the number of syllables from the specific song-recording file found to contain the yellow-purple transition by the clustering procedure was 13 while manual scoring included only 11, illustrating and accounting for the transition probability discrepancy between the two analyses (Figure 3).

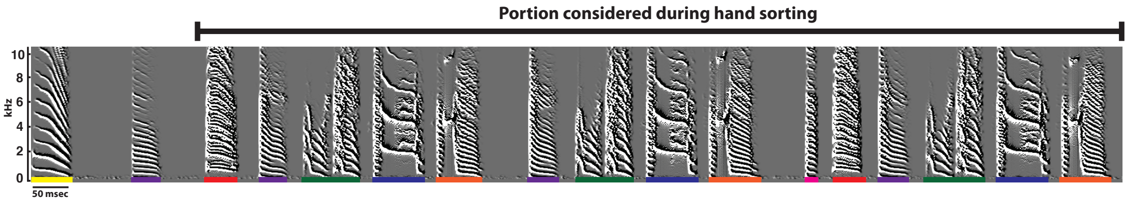

Figure 3 I Determination of transition discrepancy between VoICE and human scoring results from difference of opinion between the onset of a singing bout. Colors indicate cluster assignments as determined by VoICE.

The weighted unpenalized syntactical similarity between transition probability matrices created from the semi-automated clustering results and the data scored by hand was 0.9994, indicating nearly identical syntaxes were identified by the two scoring methods.
